# The 3*S* Enantiomer Drives Enolase Inhibitory Activity in SF2312 and Its Analogues

**DOI:** 10.3390/molecules24132510

**Published:** 2019-07-09

**Authors:** Federica Pisaneschi, Yu-Hsi Lin, Paul G. Leonard, Nikunj Satani, Victoria C. Yan, Naima Hammoudi, Sudhir Raghavan, Todd M. Link, Dimitra K. Georgiou, Barbara Czako, Florian L. Muller

**Affiliations:** 1 Department of Cancer Systems Imaging, University of Texas MD Anderson Cancer Center, Houston, TX 77054, USA; 2 Institute of Applied Cancer Science, University of Texas MD Anderson Cancer Center, Houston, TX 77054, USA; 3 Core for Biomolecular Structure and Function, Department of Genomic Medicine, University of Texas MD Anderson Cancer Center, Houston, TX 77054, USA

**Keywords:** glycolysis, enolase, chiral, phosphonate, hydroxamate, *E. coli*, enzyme inhibitor, enzyme structure, natural product, X-ray crystallography

## Abstract

We recently reported that SF2312 ((1,5-dihydroxy-2-oxopyrrolidin-3-yl)phosphonic acid), a phosphonate antibiotic with a previously unknown mode of action, is a potent inhibitor of the glycolytic enzyme, Enolase. SF2312 can only be synthesized as a racemic-diastereomeric mixture. However, co-crystal structures with Enolase 2 (ENO2) have consistently shown that only the (3*S*,5*S*)-enantiomer binds to the active site. The acidity of the alpha proton at C-3, which deprotonates under mildly alkaline conditions, results in racemization; thus while the separation of four enantiomeric intermediates was achieved via chiral High Performance Liquid Chromatography (HPLC) of the fully protected intermediate, deprotection inevitably nullified enantiopurity. To prevent epimerization of the C-3, we designed and synthesized MethylSF2312, ((1,5-dihydroxy-3-methyl-2-oxopyrrolidin-3-yl)phosphonic acid), which contains a fully-substituted C-3 alpha carbon. As a racemic-diastereomeric mixture, MethylSF2312 is equipotent to SF2312 in enzymatic and cellular systems against Enolase. Chiral HPLC separation of a protected MethylSF2312 precursor resulted in the efficient separation of the four enantiomers. After deprotection and inevitable re-equilibration of the anomeric C-5, (3*S*)-MethylSF2312 was up to 2000-fold more potent than (3*R*)-MethylSF2312 in an isolated enzymatic assay. This observation strongly correlates with biological activity in both human cancer cells and bacteria for the 3*S* enantiomer of SF2312. Novel X-ray structures of human ENO2 with chiral and racemic MethylSF2312 show that only (3*S,*5*S)*-enantiomer occupies the active site. Enolase inhibition is thus a direct result of binding by the (3*S,*5*S)*-enantiomer of MethylSF2312. Concurrent with these results for MethylSF2312, we contend that the (3*S*,5*S*)-SF2312 is the single active enantiomer of inhibitor SF2312.

## 1. Introduction

Glycolysis is a conserved catabolic pathway [1], with a set of essential glycolysis genes and corresponding enzymes present in most organisms [2,3]. Given that glycolytic deregulation has been implicated in a number of diseases, such as cancer [4,5,6,7], malaria [8,9], and Trypanosoma [10,11], makes the rarity of natural product inhibitors of glycolysis particularly striking. Within the context of cancer, many tumors exhibit a shift in metabolism, favoring glycolysis, in what is known as the Warburg Effect [12]. Despite the clinical significance of inhibiting glycolytic enzymes, few high-affinity glycolysis inhibitors have been described, with most existing as tool compounds with limited translatable utility [13]. Even fewer is the number of natural antibiotic inhibitors of this pathway.

Though cancer cells may exhibit the Warburg Effect, one major caveat to inhibiting glycolysis is that general inhibition does not provide a sufficiently large therapeutic window for anti-neoplastic activity. Capitalizing on this hallmark feature of cancers while maintaining a sufficient therapeutic window, thus, presents a clinically-useful paradigm. In concurrence, we have recently described a novel therapeutic strategy termed “collateral lethality [4,5],” wherein metabolic vulnerabilities are endowed by the concomitant loss of tumor suppressor genes and neighboring housekeeping genes with vital but redundant function. Proof-of-principle studies were conducted on tumors bearing homozygous deletions of the 1p36 tumor suppressor locus, which results in loss of the enzyme Enolase 1 (ENO1). Enolase is a key enzyme involved in the penultimate step of glycolysis, catalyzing the conversion of 2-phosphoglycerate (2-PGA) to phosphoenolpyruvate (PEP). Tumors harboring homozygous deletion of ENO1 remain viable through the action of its functionally redundant paralog, ENO2. Inhibition of ENO2 thus results in selective killing of ENO1-null cancers [4].

**Figure 1:**
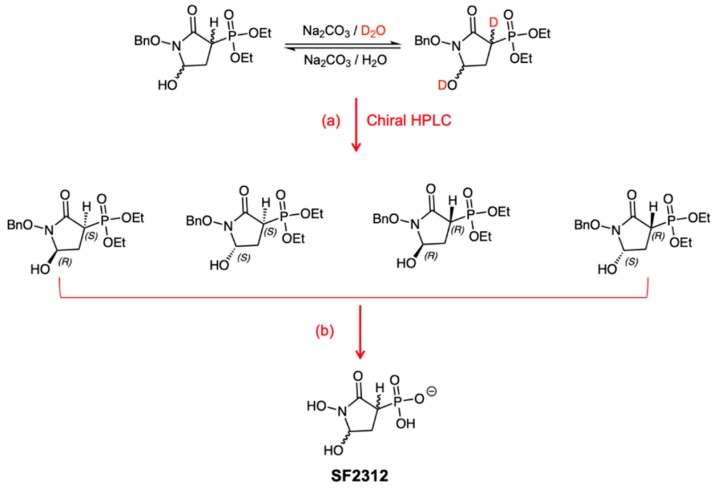
In racemic, protected SF2312, H-3 is acidic with a predicted pKa = 7.7 and exchanges with deuterium when treated with D_2_O and sodium carbonate. After separation by chiral HPLC, four enantiomers were separated, but they quickly epimerize to form racemic SF2312 as a diastereomeric mixture. Reagents and conditions: (a) Lux Cell-1 21.2 × 150 mm (Phenomenex, Torrence, CA, USA) column. (b) 1. TMSBr, DCM, rt, overnight (60%); 2. Pd(OH)_2_, water, 4 h, rt (60%).

To therapeutically exploit this vulnerability, we recently reported the synthesis and activity of the natural phosphonate antibiotic, SF2312—the most potent natural inhibitor of glycolysis reported to date [14]. SF2312 is a transition state analogue that inhibits Enolase at low nanomolar concentrations. Structurally, SF2312 has two stereocenters. However, X-ray co-crystal structures with ENO2 show that only the (*S*,*S*)-enantiomer occupies the active site [14] (PDB: 4ZCW). Therefore, we sought to separate individual enantiomers to validate the exclusivity of inhibition by the (*S*,*S*)-enantiomer of SF2312. Our initial attempts by chiral HPLC proved challenging due to the absence of UV-active moieties on the molecule. To amend this issue, we sought to isolate individual isomers of a synthetic precursor, diethyl (1-(benzyloxy)-5-hydroxy-2-oxopyrrolidin-3-yl)phosphonate (Figure 1), as the *O*-benzyl protecting group is visible at 254 nm. Indeed, the separation of the four isomers was successful. But subsequent reactions towards the final product nullified enantiopurity: under alkaline conditions, both the C-5 anomeric hemiaminal and the C-3 alpha carbon spontaneously epimerize [14] (Figure 1). This is due to the acidity of the alpha proton, which has a predicted pKa of 7.7 (prediction via Chemicalize). When placed in D_2_O, with catalytic sodium bicarbonate, we observed deuteron exchange at the alpha carbon and the alcohol at C-5.

**Figure 2:**
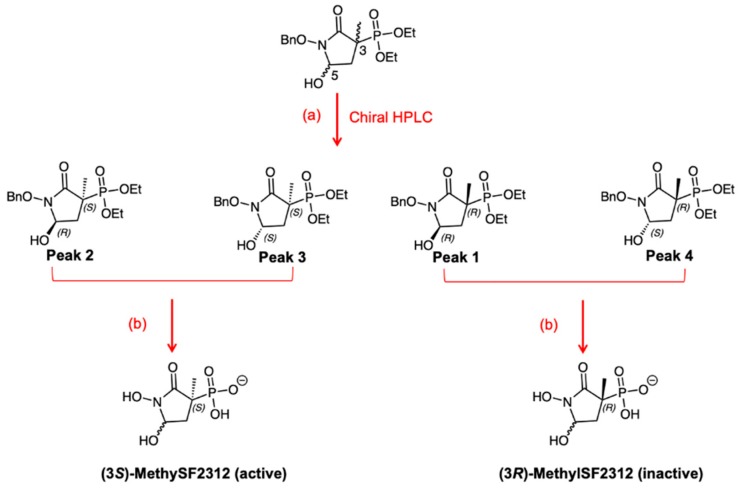
In racemic, protected MethylSF2312, C-3 is blocked and cannot epimerize. After separation by chiral HPLC, four enantiomers were separated. C-5 epimerizes during the deprotection reactions, affording chirally active (3*S*)- and (3*R*)-MethylSF2312 as diastereomeric mixtures. Reagents and conditions: (a) Lux Cell-1 21.2 × 150 mm (Phenomenex, Torrence, CA, USA) column. (b) 1. TMSI, DCM, rt, overnight (40%); 2. Pd(OH)_2_, water, 4 h, rt (75%).

**Figure 3:**
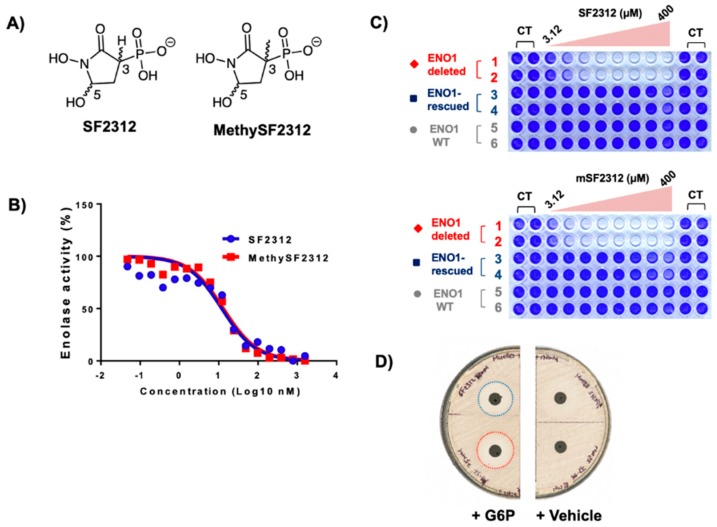
MethylSF2312 exhibits the same Enolase inhibitory potency and biological activity as SF2312. (**A**) Structures of SF2312 and MethylSF2312; (**B**) Enolase inhibitory activity (*x*-axis Enolase activity, *y*-axis, inhibitor concentration nM) of racemic SF2312 and MethylSF2312 in vitro using lysates of *E. coli* cells (blue: SF2312, red: MethylSF2312). (**C**) Sensitivity of D423 ENO1-deleted (red), D423 ENO1-rescued (blue) and LN319 ENO1-WT (grey) glioma cells to racemic SF2312 (upper panel) and MethylSF2312 (lower panel). (**D**) Sensitivity of *Escherichia coli* to racemic SF2312 and MethylSF2312; zone of inhibition on a lawn of *E. coli* 0.2 μmoles of SF2312 (white dashed circle) and MethylSF2312 (red dashed circle). Glucose 6-phosphate (G6P) was added to induce expression of the phosphor-hexose transporter allowing uptake of phosphonates.

Here, we report the synthesis of the 3-methyl derivative of SF2312: MethylSF2312 (1,5-dihydroxy-3-methyl-2-oxopyrrolidin-3-yl)phosphonic acid). Methyl substitution at C-3 renders a fully substituted alpha carbon, which preserves stereochemistry after chiral separation (graphically illustrated in Figure 2). We show that MethylSF2312 is similar to SF2312 for inhibiting Enolase both in vitro and in cell-based systems (Figure 3). Co-crystallization of MethylSF2312 with ENO2 shows that only the (3*S*,5*S*)-enantiomer is the active species. This upholds its role as a suitable analogue for examining the role of chirality in active site Enolase inhibition, especially for its use as an anticancer agent against *ENO1*-deleted glioblastoma.

## 2. Results and Discussion

### 2.1. MethylSF2312 Is a Potent Enolase Inhibitor with a Non-Epimerizable (3S,5S) Stereocenter

**Scheme 1:**
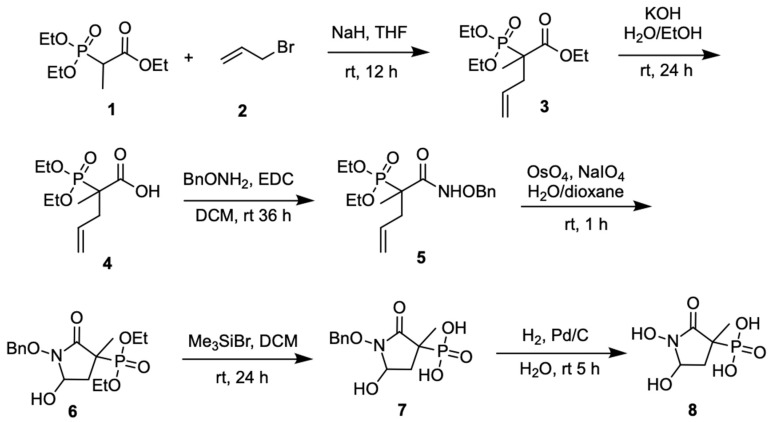
Synthesis of MethylSF2312.

Both SF2312 and MethylSF2312 were synthesized according to previously published procedures [15]; triethyl 2-phosphonopropionate (**1**) was used as the starting material for the latter, rather than triethyl phosphonoacetate (Scheme 1). SF2312 and MethylSF2312 were obtained as a racemic mixture of cis and trans diastereomers in ~1:1 ratio.

To compare the effects of isomeric mixtures of MethylSF2312 and SF2312 on Enolase activity, we measured the formation PEP coupled to the oxidation of NADH via LDH/PK on human cancer cells overexpressing ENO2 [4,14]. Both SF2312 and MethylSF2312 had an IC_50_ of ~10 nM for ENO2. We then used a second assay that directly measures PEP formation at 240 nm in *Escherichia coli* lysates [13] to substantiate our initial findings. Here, we favored the direct measurement of PEP absorbance, rather than of NADH, due to the high levels of endogenous NADH oxidase activity in bacterial lysates. Again, we found that both SF2312 and MethylSF2312 had an IC_50_ of ~10 nM (Figure 3B).

We then examined biological toxicity in two cell-based systems. Our earlier reports show that *ENO1*-homozygous deleted glioma cells are exquisitely sensitive to SF2312, compared to ENO1 isogenic rescued cells and ENO1-intact glioma control cells [14]. Encouraged by our initial in vitro results, we sought to determine whether similar results could be obtained with MethylSF2312. Indeed, after six days of treatment, both SF2312 and MethylSF2312 exhibited selective toxicity against ENO1-deleted versus *ENO1*-intact glioma cells. MethylSF2312 displayed equipotency and, in some cases, even slightly exceeded the potency of SF2312 (Figure 3C).

Finally, the antibiotic activities of SF2312 and MethylSF2312 were compared in *E. coli* using the disc diffusion method [16]. A 7 mm filter paper disc imbued with 35 µg of either SF2312 or MethylSF2312 was placed on an *E. coli* lawn on Muller-Hinton Agar. Overnight incubation resulted in a ~24 mm clear zone of growth inhibition, which was essentially the same for SF2312 and MethylSF2312 (Figure 3D). Of note, growth inhibition was only observed with the addition of glucose-6-phosphate (G6P). Similar to other phosphonate antibiotics such as Fosfomycin, this indicates that SF2312 and its derivatives induce the bacterial hexose-6-phosphate transporter to gain cell entry [17].

Together, our data from distinct organisms converge on the idea that MethylSF2312 is sufficiently analogous to SF2312 for elucidating the proper stereochemistry involved in active site binding, while maintaining similar levels of inhibitory potency.

### 2.2. Chiral Fractions of MethylSF2312 Show Dramatic Differences in Enolase Inhibitory Activity

**Figure 4:**
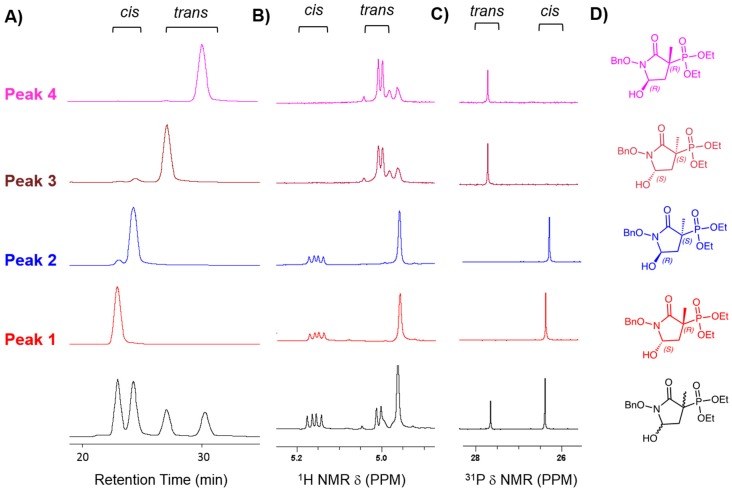
Chiral separation of MethylSF2312 precursor into four enantiopure fractions. (**A**) Chromatogram of fully protected MethylSF2312 (Intermediate **6**, diethyl (1-(benzyloxy)-5-hydroxy-3-methyl-2-oxopyrrolidin-3-yl)phosphonate), consisting of a racemic mixture of *cis*/*trans* isomers in a ~2:1 ratio (black trace, *x*-axis shows retention time and *y*-axis absorption at 254 nm). Enantiomeric purity was verified by re-running each fraction on the same column (red, blue, brown, pink traces. Peak 1 (P1, rt = 23 min) showed 99.9% enantiomeric purity, peak 2 (P2, rt = 24.2 min) 96%, peak 3 (P3, rt = 27.1 min) 95% and peak 4 (P4, rt = 30.2 min) 97%. These were assigned to the *cis* and *trans* isomers based on Nuclear Magnetic Resonance (NMR). (**B**) Benzylic and 5-H protons; (**C**) ^31^P peaks*; cis*/*trans* isomers of intermediate **6** were assigned by NOESY with reference to [5]. (**D**) Structures of the chiral fractions. Structure of fraction 2 was assigned the *S* configuration based on the X-ray structure and the configuration of the other stereocenters was deduced thereafter from the cis/trans isomers (see results).

High polarity and the absence of UV-detectable moieties render SF2312 and MethylSF2312 difficult to resolve by chiral chromatography. As such, their benzyl-protected, racemate esters were selected for chiral purification due to their added hydrophobicity. The MethylSF2312 precursor diethyl (1-(benzyloxy)-5-hydroxy-3-methyl-2-oxopyrrolidin-3-yl)phosphonate **6** (Scheme 1) was separated on a Lux-Cel chiral HPLC column (Phenomenex). Intermediate **6** started as a racemic mixture of cis/trans isomers in a ~2:1 ratio. After column purification, the chromatogram showed four distinct peaks (fractions): two major and two minor, in a ratio of ~2:1. The four fractions were then separated, and each was analytically re-run through the same column to evaluate enantiopurity (Figure 4A), at which point each pure fraction yielded a single major peak. In analyzing the chromatograms of each fraction, we found that the first compound (P1, peak 1) showed the greatest enantiopurity. The other three fractions (P2, P3, P4; peak 2, peak 3, peak 4, respectively) showed some level of cross-contamination, which was likely due to tailing.

**Figure 5:**
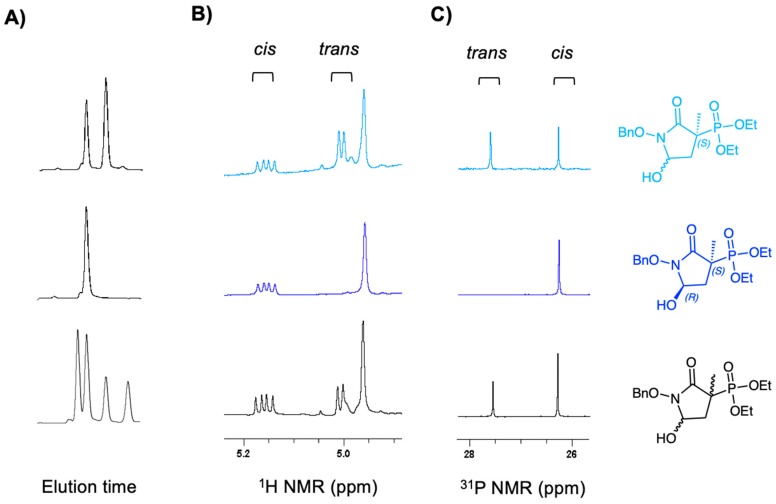
Epimerization of MethylSF2312 precursor **6** in alkaline media. (**A**) Chromatograms, (**B**) ^1^H NMR spectra of benzylic and hemiaminal protons of (*x*-axis ppm), and (**C**) ^31^P (proton decoupled) spectra of the same preparations, with chiral structural assignments based on the X-ray structure of the final product. *Bottom panel*: racemic compound **6**; *Middle panel*: enantiopure (3*S*,5*R*)-**6**; top panel: (3*S*,5*SR*)-**6**, obtained after treatment of (3*S*,5*R*)-**6** with sodium bicarbonate.

Immediately after MethylSF2312 enantiomers were collected from the column, we performed NMR analysis to mitigate water exposure towards the anomeric equilibration. A single drop of deuterated acetonitrile was added for signal lock. The diastereomeric purity (99.9%) of compound P1 was confirmed by ^1^H and ^31^P NMR (Figure 4B,C), while the other fractions (P2–4) proved to be highly enantioenriched (95–97%). Though NMR cannot determine absolute stereochemistry, it can readily distinguish cis/trans isomers. Thus, while the racemic MethylSF2312 starting mix showed two peaks by ^31^P NMR, which correspond to the cis and trans isomers in ~2:1 ratio, each enantiopure fraction yielded only a single major ^31^P NMR peak. P1 and P2 were identical while P3 and P4 were identical by ^31^P and ^1^H NMR. 2D NOESY spectroscopy indicated that P1 and P2 corresponded to the cis diastereomer, while P3 and P4 corresponded to the trans diastereomer. To highlight the base-labile nature of the C5 proton on MethylSF2312, we added a saturated solution of NaHCO_3_, which expectedly resulted in epimerization and generation of cis/trans diastereomers (Figure 5). Indeed, NMR cannot determine the absolute chiral configuration. However, when coupled with our co-crystal structure data showing MethylSF2312 in the 3*S* configuration with ENO2 (see below), we can deduce the absolute configuration of each enantiopure fraction. Analysis by NOESY NMR showed that P2 was a *cis* isomer. Upon assignment of its absolute configuration at C3, we can readily deduce that P2 must be the (3*S*,5*S*)-isomer. Treatment with a base causes P2 to epimerize into P2 and P3, which confirms P3 as the trans-(3*S*,5*R*) isomer. In turn, P1 and P4 can only be the *cis*-(3*R*,5*R*) and *trans*-(3*R*,5*S*), respectively.

MethylSF2312 was obtained by full deprotection of the phosphonate and hydroxamate moieties. Chiral fractions, P1 and P2, were advanced to biochemical testing because these showed the highest level of enantiopurity. Deprotection inevitably yielded equilibration of the anomeric C-5. (3*R*)-MethylSF2312, derived from P1, as expected, was indistinguishable by NMR from (3*S*)-MethylSF2312 derived from P2. To substantiate the identity of these compounds, we employed high-resolution mass spectrometry (HRMS). The expected HRMS (ES-) mass for C_5_H_10_NO_6_P [M − H]^−^ expected mass is 210.0173. For the initial racemic mixture of MethylSF2312, the observed [M − H]^−^ mass was 210.0178. For P1-derived (*3R*)-MethylSF2312 it was 210.0180. For P2-derived (*3S*)-MethylSF2312 it was 210.0182. These multiple avenues of evidence confirmed that the chemical formulas for P1 and P2-derived MethylSF2312 were identical.

**Figure 6:**
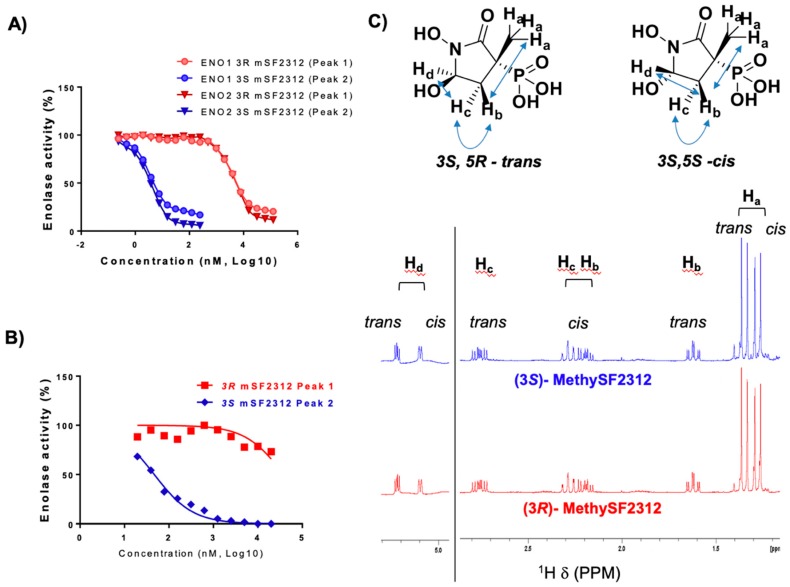
Dramatic difference in Enolase inhibitory potency between MethySF2312 generated from different chiral fractions. Enantiopure fractions were taken through deprotection reactions to remove the ethyl ester and benzyl protecting groups and subsequently tested for Enolase inhibitory activity. (**A**) Enolase activity in lysates from ENO1 (circles) and ENO2 (diamonds) D423 cells as a function of inhibitor concentration. (3*S*)-MethylSF2312 in blue and light blue; (3*R*)-MethylSF2312 in red and light red). (**B**) Enolase activity in lysates of *E. coli*. (3*S*)-MethylSF2312 in blue and (3*R*)-MethylSF2312 in red. (**C**) ^1^H NMR spectra of (3*R*)- and (3*S*)-MethylSF2312 in D_2_O, with proton assignments. The structure of cis/trans isomers and NOESY interactions are shown.

We then determined Enolase inhibitory activity of (*3R*)-P1 and (*3S*)-P2 derived MethylSF2312 in native lysates from human cancer cells and *E. coli* overexpressing ENO1 and ENO2. Against ENO1 and ENO2, (*3S*)-MethylSF2312 had an IC_50_ of ~10 nM. In sharp contrast, (*3R*)-MethylSF2312 proved to be 2000-fold less potent, with an IC_50_ of ~15 µM. Similar results were obtained for enolase inhibition in *E. coli* lysates: (*3S*)-MethylSF2312 exhibited ~2000-fold greater Enolase inhibitory potency compared to (*3R*)-MethylSF2312 (Figure 6).

Whether (*3R*)-MethylSF2312 genuinely retains any Enolase inhibitory activity or whether the observed inhibition (>15 µM) is due to trace (>0.05%) contamination by P2 is not entirely clear. While the enantiopurity by chiral HPLC of P1 is greater than 99%, we certainly cannot rule out the possibility of micro-contamination. In light of our results, we see that the best interpretation is that (*3S*)-MethylSF2312 is the single isomer with Enolase inhibitory activity. This was further supported by the X-ray structure.

### 2.3. The Biological Activity of Each Chiral MethylSF2312 Fraction Strongly Correlates with Enolase Inhibitory Activity

**Figure 7:**
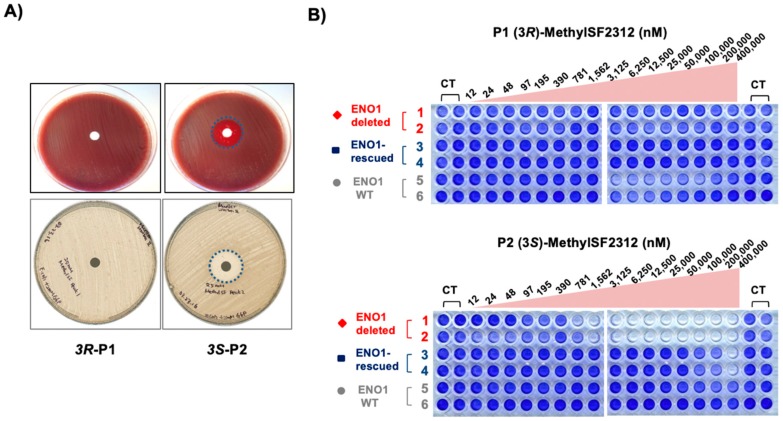
Biological activity correlates with Enolase inhibitory activity of MethylSF2312 chiral fractions. (**A**) Each disc was imbued with 35 μg of either (3*S*)- or (3*R*)-MethylSF2312 and incubated overnight at 37 °C on a lawn of *E. coli* in Columbia blood agar (top) or Muller Hinton agar (bottom) with G6P. Zones of clearing are shown by dashed blue circles. (**B**) D423 ENO1 deleted (red), D423 isogenic rescued (blue) and LN319 ENO1-WT (grey) were treated with (3*R*)- or (3*S*)-MethylSF2312 for 7 days, and cell density quantified by crystal violet.

We determined the effects of MethylSF2312 derived from (*3R*)- and (*3S*)-enantiomers on mammalian glioma cells and *E. coli* in culture. Biological activity closely mirrored in vitro Enolase inhibitory potency. Concurrent with our initial findings, (*3R*)-MethylSF2312 displayed minimal toxicity towards *ENO1*-deleted glioma cells, even at 400 µM. In stark contrast, (*3S*)-MethylSF2312 exhibited toxicity to *ENO1*-deleted glioma cells at ~2 µM (Figure 7), which is similar to that observed for racemic SF2312 in our previous work (Figure 3) [4,14]. Selectivity for *ENO1*-deleted over *ENO1*-intact glioma cells is maintained (Figure 7).

We then repeated this experiment in *E. coli* using the disc-diffusion method of antibiotic sensitivity testing. Here, only the *S*-enantiomer generated a distinct zone of inhibition, which was evident in both Muller Hinton Agar as well as Blood Agar (Figure 7). In sum, these data reinforce the notion that meaningful biological activity is restricted to the (*3S*)-enantiomer of MethylSF2312.

### 2.4. X-ray Structures of ENO2 are Only Occupied by the S-enantiomer of MethylSF2312

MethylSF2312 was co-crystallized with Human ENO2 to determine the configuration of the active compound in the active site. Apo crystals of ENO2 were prepared using published methods [14] and were soaked in cryoprotectant containing the racemic mixture of MethylSF2312 for 16 h prior to freezing. The resulting co-crystal structure was resolved (Table 1), with four ENO2 homodimers in the asymmetric unit of the monoclinic crystal (PDB 5EU9).

**Figure 8:**
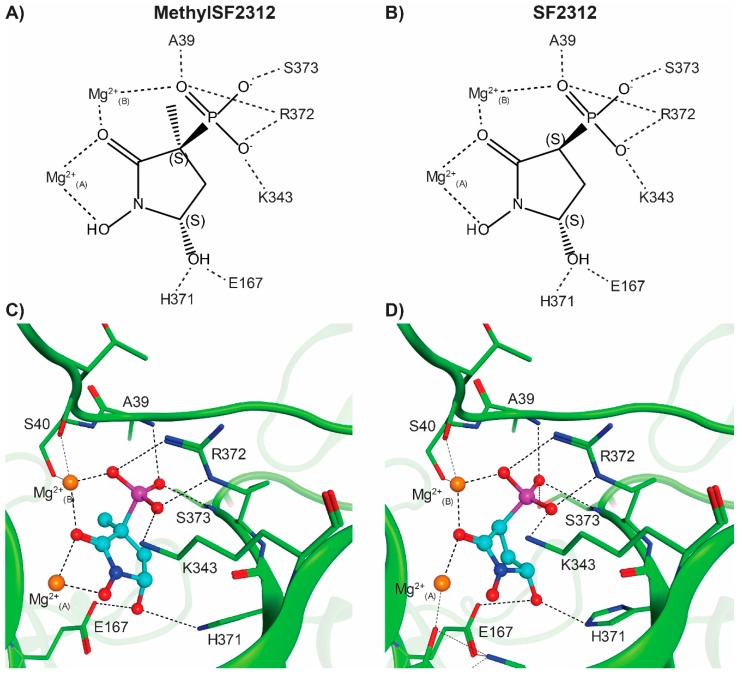
Racemic MethylSF2312 and SF2312 bind ENO2 in the 3*S*,5*S* configuration. A schematic depiction (**A**,**B**) and a three dimensional view (**C**,**D**) of the ENO2:MethylSF2312 structure (PDB: 5EU9, (**A**,**C**)) and ENO2:SF2312 (PDB: 4ZCW, (**B**,**D**)) showing the direct interaction with the ENO2 protein and the Magnesium ions in the active site. The hydrogen bonds, metal coordination bonds and salt bridge interactions are indicated by dashed lines. The two magnesium ions and water molecules in the active site pocket are represented by orange and red spheres, respectively. Although both ENO2 crystals were soaked with racemic SF2312 and MethylSF2312, in each case, only the (3*S*,5*S*)-enantiomer could be fitted to the electron density map. All major H-bonds and backbone interactions are essentially the same for SF2312 and MethylSF2312.

**Figure 9:**
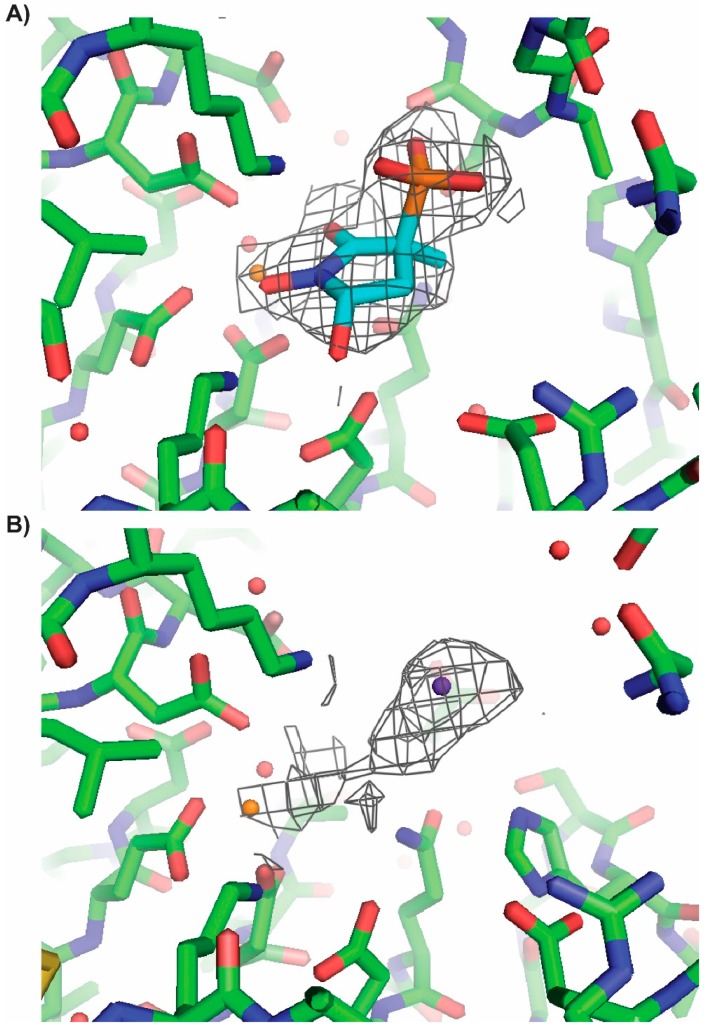
P2 derived MethylSF2312 binds in the 3S conformation. ENO2 crystals were soaked with MethylSF2312 derived from P2 (Panel **A**, PDB: 5TIJ) or P1 (Panel **B**, PDB: 5TD9). An unbiased 2Fo-Fc omit electron density map with a cutoff of 1σ is depicted as a grey mesh. The electron density map confirms that ENO2 soaked with P2-derived MethylSF2312 is occupied in 3*S*,5*S*-configuration (Panel **A**) while there is no evidence of occupancy for ENO2 soaked with P1-derived (3*R*)-MethylSF2312 (Panel **B**). Magnesium and chloride atoms are shown in orange and purple, respectively.

We observed only the 3*S* enantiomer of MethylSF2312 interacting with both the conformational and catalytic magnesium ions (Mg^2+^_(A)_ and Mg^2+^_(B)_ respectively) in all eight active sites in the crystal asymmetric unit (Figure 8 and Figure 9), despite incubating with a racemic mixture. These observed key interactions are consistent with our previous reports on the binding mode of SF2312 (Figure 8) even with the enhanced planarity of the pyrrolidine ring when a methyl group is present at the 3′ position.

The phosphonate moiety proves to be critically involved in active site interaction. In addition to forming hydrogen bonds with the backbone amides of Gly38 and Ala39, it also forms a salt bridge with Arg372 and directly interacts with the catalytic magnesium ion (Mg^2+^_(B)_). Moving to the center of the molecule, the carbonyl oxygen sits between two magnesium ions, contributing to the octahedral coordination interaction around each magnesium. The 1′-hydroxyl group completes the octahedral coordination around the conformational magnesium (Mg^2+^_(A)_). Finally, the 5′-hydroxyl forms the hydrogen bonds with the polar sidechains of Glu167 and His371, with a strong preference for the *5S*-enantiomers of SF2312 and MethylSF2312. For further confirmation that the 3*S*-enantiomer is the active inhibitor, purified (*3R*)- and (*3S*)-MethylSF2312 were soaked into apo ENO2 crystals. With (*3R*)-MethylSF2312 (PDB:5TD9), we did not observe electron density consistent with either the 3*R*- or 3*S*- enantiomer of MethylSF2312 in the active site pocket. Also, dissimilar was the open conformation of the Pro36 to Leu47 loop, which was about 12 Å further from the active site pocket compared to that observed for the MethylSF2312 bound structure (PDB:5EU9). Conversely, apo ENO2 crystals soaked in cryoprotectant containing the active (*3S*)-MethylSF2312, unambiguously placed the 3*S*-enantiomer into the electron density map (Figure 8 and Figure 9). To avoid any model bias effects, an unbiased omit map of the active site pocket was calculated from the refined crystal structures, with the coordinates for waters, the chloride ion, and MethylSF2312 removed from the respective structure coordinate file (Figure 8). This unbiased omit map substantiates that the *3S*-enantiomer of MethylSF2312 is the active inhibitor. There is no evidence of an interaction between the 3*R*-enantiomer and ENO2.

MethylSF2312 derived from P2 (Panel A, PDB: 5TIJ) or P1 (Panel B, PDB: 5TD9). An unbiased 2Fo-Fc omit electron density map with a cutoff of 1σ is depicted as a grey mesh. The electron density map confirms that ENO2 soaked with MethylSF2312 P2 is occupied in 3S,5S conformation (Panel A) while there is no evidence of occupancy for ENO2 soaked with (3*R*)-MethylSF2312 (Panel B). Magnesium and chloride atoms are shown in orange and purple, respectively.

## 3. Materials and Methods

### 3.1. Chemistry

All reagents, for which syntheses are not described, were commercially available and have been used without any further purification, if not specified otherwise. ^1^H, ^31^P, and ^13^C NMR determinations were performed at M.D. Anderson’s NMR Core, using the Bruker 300, 500, and 600 MHz instruments as indicated. Chemical shifts were reported in parts per million (ppm) relative to the residual solvent peak rounded to the nearest 0.01 for proton and 0.1 for carbon. Coupling constants (*J*) were reported in Hz to the nearest 0.01 Hz. Peak multiplicity was indicated as follows s (singlet), d (doublet), t (triplet), q (quartet), m (multiplet), and br (broad signal). The high-resolution mass spectrometry was acquired by the Baylor University Metabolomics core, as a fee for service. Compounds were run by LC-MS in reverse phase, using a gradient of 0.1% acid in water and 0.1% formic acid in acetonitrile on an Agilent XDB C18 HPLC column and resolved by QTOF MS. MethylSF2312 had a retention time of ~0.45 min.

#### 3.1.1. Synthesis of SF2312

SF2312 was synthesized according to previously published procedures [15].

#### 3.1.2. Synthesis of MethylSF2312

*Ethyl 2-(diethoxyphosphoryl)-2-methylpent-4-enoate* (**3**): To a solution of ethyl 2-(diethoxyphosphoryl)propanoate (**1**, 10.0 g, 42 mmol) in THF (100 mL) at 0 °C, NaH (2 g, 50 mmol) was added. The mixture was stirred at 0 °C for 1 h. Then 3-bromoprop-1-ene (**2**, 6.1 g, 50 mmol) was added. The mixture was stirred at RT overnight. NH_4_Cl_aq_ (50 mL) was added at 0 °C, THF was removed and the mixture extracted with DCM (3 × 200 mL). The combined organic layers were dried over anhydr-MgSO_4_, filtered and concentrated to give compound **3** as yellow oil (12 g, 100% yield) which was used in the next step without further purification. MS (ES+) C_12_H_23_O_5_P requires: 278, found: 279.

**3:** [M + H]^+^; ^1^H NMR (600 MHz, CDCl_3_) δ 5.74–5.62 (m, 1H; 4-H), 5.15–5.07 (m, 2H, 5-H), 4.23–4.12 (m, 6H; OC*H_2_*CH_3_), 2.91 (ddd, *J* = 13.3, 10.3, 6.8 Hz, 1H; 3_a_-H), 2.40 (dddd, *J* = 13.8, 9.0, 8.0, 1.0 Hz, 1H; 3_b_-H), 1.41 (d, *J* = 16.5 Hz, 3H; 2-CH_3_), 1.33 (t, *J* = 7.1 Hz, 6H; OCH_2_C*H_3_*), 1.28 (td, *J* = 7.1, 0.8 Hz, 3H; OCH_2_C*H_3_*); ^13^C NMR (151 MHz, CDCl_3_) δ 171.1 [*s*, (d, *J* = 3.8 Hz); C-1], 132.5 [*d*, (d, *J* = 13.8 Hz); C-4], 119.2 (*t*; C-5), 63.0 [*t*, (d, *J* = 7.1 Hz); O*C*H_2_CH_3_], 62.7 [*t*, (d, *J* = 7.2 Hz); O*C*H_2_CH_3_], 61.4 (*t*; O*C*H_2_CH_3_), 48.0 [*s*, (d, *J* = 134.3 Hz); C-2], 38.4 [*t*; (d, *J* = 3.8 Hz); C-3], 17.2 [*q*; (d, *J* = 4.6 Hz); *C*H_3_-2], 16.5 [*q*, (d, *J* = 4.1 Hz); OCH_2_*C*H_3_], 16.4 [*q*, (d, *J* = 4.1 Hz); OCH_2_*C*H_3_], 14.1 (*q*; OCH_2_*C*H_3_).

*2-(Diethoxyphosphoryl)-2-methylpent-4-enoic acid* (**4**)*:* To a solution of ethyl 2-(diethoxyphosphoryl)-2-methylpent-4-enoate (**3**, 12 g, 42 mmol) in EtOH (100 mL) 1 M aq. LiOH (65 mL, 65 mmol) was added. The mixture was stirred at RT for 48 h. Then the mixture was heated to 55 °C for 48 h. The solvent was removed, the mixture was diluted with water (50 mL), extracted with DCM (2 × 100 mL). The pH was then adjusted with 1M HCl, and the mixture extracted with DCM (3 × 100 mL). The combined organic layers were dried over anhydr-MgSO_4_, filtered and concentrated to yield the title compound **4** as a yellow oil (8 g, 76% yield), which was used in the next step without further purification.

**4:** MS (ES+) C_10_H_19_O_5_P requires:250, found: 251 [M + H]^+^; ^1^H NMR (600 MHz, CDCl_3_) δ 5.76 (dddd, *J* = 16.8, 10.2, 7.5, 6.2 Hz, 1H; 4-H), 5.25–5.01 (m, 2H; 5-H), 5.03 (br s, 1H; OH), 4.29–4.17 (m, 4H; OC*H_2_*CH_3_), 2.94–2.83 (m, 1H; 3_a_-H), 2.45–2.36 (m, 1H; 3_b_-H), 1.42 (d, *J* = 16.5 Hz, 3H; 2-CH_3_), 1.37 (t, *J* = 6.9 Hz, 3H; OCH_2_C*H_3_*), 1.36 (t, *J* = 6.9 Hz, 3H; OCH_2_C*H_3_*); ^13^C NMR (151 MHz, CDCl_3_) δ 172.6 [s, (d, *J* = 2.6 Hz); C-1], 132.3 [ d, (d, *J* = 13.1 Hz); C-4), 119.2 (t; C-5), 63.7 [t, (d, *J* = 7.2 Hz); O*C*H_2_CH_3_], 63.2 [t; (d, *J* = 7.4 Hz); O*C*H_2_CH_3_], 47.9 [s, (d, *J* = 134.1 Hz); C-2], 38.5 [t; (d, *J* = 4.1 Hz); C-3), 17.1 [q, (d, *J* = 4.4 Hz); OCH_2_*C*H_3_], 16.4 [q, (d, *J* = 5.9 Hz); OCH_2_*C*H_3_], 16.3 [q, (d, *J* = 5.7 Hz); OCH_2_*C*H_3_].

*Diethyl 1-(benzyloxyamino)-2-methyl-1-oxopent-4-en-2-ylphosphonate* (**5**): To a solution of 2-(diethoxyphosphoryl)-2-methylpent-4-enoic acid (**4**, 8.0 g, 32 mmol) and *O*-benzylhydroxylamine hydrochloride (4.3 g, 35.2 mmol) in DCM (200 mL) DMAP (5.9 g, 48 mmol) and EDC hydrochloride (6.2 g, 48 mmol) were added. After stirring for 36 h at RT, the mixture was washed with 1M HCl (2 × 50 mL) and brine (50 mL). The organic layer was dried over anhydr-MgSO_4_ and evaporated in vacuum to obtain compound **5** as yellow oil (9 g, 79% yield), which was used in the next step without further purification.

**5:** MS (ES+) C_17_H_26_NO_5_P requires: 355, found: 356 [M + H]^+^; ^1^H NMR (600 MHz, CDCl_3_) δ 9.72 (s, 1H; NH), 7.43–7.31 (m, 5H; Ph), 5.75–5.63 (m, 1H; 4-H), 5.14–5.05 (m, 2H; 5-H), 4.93 (A of AB system, *J* = 11.2 Hz, 1H; C*H*HPh), 4.88 (B of AB system, *J* = 11.1 Hz, 1H; C*H*HPh), 4.18–3.96 (m, 4H; OC*H_2_*CH_3_), 2.71–2.60 (m, 1H; 3_a_-H), 2.40–2.32 (m, 1H; 3_b_-H), 1.34 (d, *J* = 15.6 Hz, 3H; 2-CH_3_), 1.32 (t, *J* = 7.1 Hz, 3H; OCH_2_C*H_3_*), 1.27 (t, *J* = 7.1 Hz, 3H; OCH_2_C*H_3_*); ^13^C NMR (151 MHz, CDCl_3_) δ 168.0 (*s*; C-1), 135.4(*s*; Ph), 131.9 [*d*, (d, *J* = 12.3 Hz); C-4], 129.0 (*d*, 2C; Ph), 128.6 (*d*; Ph), 128.5 (*d*, 2C; Ph), 119.3 (*t*; C-5), 78.2 (*t*; *C*H_2_Ph), 63.2 [*t*, (d, *J* = 7.3 Hz); O*C*H_2_CH_3_], 63.1 [ *t*, (d, *J* = 7.4 Hz); O*C*H_2_CH_3_], 45.9 [*s*, (d, *J* = 133.8 Hz); C-2], 39.7 [*t*, (d, *J* = 3.1 Hz); C-3], 17.0 [*q*, (d, *J* = 4.8 Hz); 2-CH_3_], 16.4 [*q*, (d, *J* = 5.7 Hz); OCH_2_*C*H_3_], 16.3 [*q*, (d, *J* = 5.7 Hz); OCH_2_*C*H_3_].

*Diethyl 1-(benzyloxy)-5-hydroxy-3-methyl-2-oxopyrrolidin-3-ylphosphonate* (**6**): To the solution of diethyl 1-(benzyloxyamino)-2-methyl-1-oxopent-4-en-2-ylphosphonate (**5**, 5.0 g, 14.1 mmol) in dioxane/H_2_O (300 mL/300 mL) was added OsO_4_ (286 mg, 1.13 mmol). The mixture was stirred at RT for 30 min, then NaIO_4_ (9 g, 42.2 mmol) was added portion-wise. The mixture was stirred at RT for 1 h, diluted with water (1000 moL) and extracted with DCM (3 × 500 mL). The combined organic layers were washed with water (300 mL) and brine (300 mL), dried over anhydr-MgSO_4_, filtered and concentrated to afford the title compound **6** as a light-yellow oil (5 g, 99% yield), which was used in the next step without further purification.

**6:** MS (ES+) C_16_H_24_NO_6_P requires: 357, found: 358 [M + H]^+^; ^1^H NMR (600 MHz, CDCl_3_) δ 7.50–4.45 (m, 5H; Ph), 7.40–7.33 (m, 5H; Ph), 5.16 (A of AB system, *J* = 10.4 Hz, 1H; C*H*HPh), 5.11 (B of AB system d, *J* = 10.4 Hz, 1H; C*H*HPh), 5.09–5.04 (m, 1H; 5-H), 5.07 (A of AB system, *J* = 10.6 Hz, 1H; C*H*HPh) 5.02 (B of AB system, *J* = 10.8 Hz, 1H; C*H*HPh), 4.97–4.89 (m, 1H; 5-H), 4.29–4.12 (m, 4H; OC*H_2_*CH_3_), 2.75 (ddd, *J* = 18.2, 14.0, 6.3 Hz, 1H; 4-H_a_), 2.42 (dd, *J* = 15.9, 14.3 Hz, 1H; 4-H_a_), 2.08–1.93 (m, 1H; 4-H_b_), 1.72 (ddd, *J* = 14.0, 12.2, 3.0 Hz, 1H; 4-H_b_), 1.61 (s, 6H), 1.54 (d, *J* = 16.8 Hz, 3H; 3-Me), 1.40 (d, *J* = 16.0 Hz, 3H; 3-Me), 1.37–1.32 (m,6H; OCH_2_C*H_3_*); ^13^C NMR (151 MHz, CDCl_3_) δ 169.6 [*s*, (d, *J* = 2.9 Hz); C-1], 167.4 [*s*, (d, *J* = 1.5 Hz); C-1], 135.1 (*s*, Ph), 134.9 (*s*, Ph), 129.6 (*d*, Ph), 129.6 (*d*, Ph), 129.0 (*s*, Ph), 128.9 (*s*, Ph), 128.6 (*d*, Ph), 128.4 (*d*, Ph), 80.5 (*d*; C-5), 80.1 [*d*, (d, *J* = 6.7 Hz); C-5], 78.2 (*t*; *C*H_2_Ph), 79.0 (*t; C*H_2_Ph), 65.3 [*t*, (d, *J* = 6.3 Hz); O*C*H_2_CH_3_], 63.6 [*t*, (d, *J* = 6.6 Hz); O*C*H_2_CH_3_], 62.7 [*t*, (d, *J* = 7.2 Hz); O*C*H_2_CH_3_], 62.6 [*t*, (d, *J* = 7.7 Hz); O*C*H_2_CH_3_], 42.0 [*s*, (d, *J* = 145.5 Hz); C-3], 41.8 [*s*, (d, *J* = 135.9 Hz); C-3], 37.7 [*t*, (d, *J* = 1.8 Hz); C-4], 35.2 [*t,* (d, *J* = 2.3 Hz); C-4], 20.5 [*q*, (d, *J* = 4.2 Hz); 3-Me], 19.3 [*q*, (d, *J* = 4.8 Hz); 3-Me], 16.5–16.4 [*q*, 3C; OCH_2_*C*H_3_], 16.3 [*q*, (d, *J* = 3.9 Hz); OCH_2_*C*H_3_]. ^31^P NMR (202 MHz, CDCl_3_) δ 27.8, 26.2.

*1-(Benzyloxy)-5-hydroxy-3-methyl-2-oxopyrrolidin-3-ylphosphonic acid* (**7**): To the solution of diethyl 1-(benzyloxy)-5-hydroxy-3-methyl-2-oxopyrrolidin-3-ylphosphonate (**6**, 5 g, 13.4 mmol) in DCM (250 mL) was added trimethylsilyl bromide (TMSBr, 8.4 g, 42 mmol). The reaction mixture was stirred at RT overnight. The mixture was then concentrated and re-dissolved in DCM (50 mL) and water (20 mL). The aqueous phase was washed with ethyl acetate (50 mL × 5). The aqueous phase was then injected into a reverse phase HPLC column to yield the title compound **7** as a white solid (1.5 g, 40%).

**7:** MS (ES+) C_12_H_16_NO_6_P requires: 301, found: 302 [M + H]^+^and 283 [M − H_2_O + H]^+^; ^1^H NMR (500 MHz, D_2_O, *cis*/*trans* mixture) δ 7.42–7.26 (m, 10 H), 5.00 (dm, *J* = 6.7 Hz, 1H; 5-H), 4.97 (dd, *J* = 6.5, 2.0 Hz, 1H; 5-H), 4.00 (A part of AB system, *J* = 7.2 Hz, 1H; C*H*HPh), 3.97 (B part of AB system, *J* = 7.2 Hz, 1H; C*H*HPh), 3.51 (A part of AB system, *J* = 7.1 Hz, 1H; C*H*HPh), 3.48 (B part of AB system, *J* = 7.1 Hz, 1H; C*H*HPh), 2.88 (ddd, *J* = 21.5, 9.6, 7.0 Hz, 1H; 4-H_a_), 2.70 (ddd, *J* = 21.2, 9.9, 4.1 Hz, 1H; 4-H_a_), 2.50–2.40 (m, 1H; 4-H_b_), 2.33–2.22 (m,1H; 4-H_b_), 1.09 (t, *J* = 7.2 Hz, 3H; Me), 1.02 (t, *J* = 7.1 Hz, 3H; Me).

*1,5-Dihydroxy-3-methyl-2-oxopyrrolidin-3-ylphosphonic acid* (MethylSF2312, **8**): To a solution of 1-(benzyloxy)-5-hydroxy-3-methyl-2-oxopyrrolidin-3-ylphosphonic acid (**7**, 100 mg; 0.33 mmol) in water (10 mL), Pd(OH)_2_ (20 mg) was added. The mixture was stirred under H_2_ atmosphere for 4 h at RT. The catalyst was then filtered, and the mixture was lyophilized to afford product **8** as white solid (50 mg, 75%).

**8:** HRMS (ES-) C_5_H_10_NO_6_P, requires: 210.0172, found: 210.0178 [M − H]^-^; ^1^H NMR (500 MHz, D_2_O, *cis*/*trans* mixture) δ 5.22 (dd, *J* = 6.9, 4.3 Hz, 1H; 5-H*_trans_*), 5.10 (dd, *J* = 6.6, 1.8 Hz, 1H; 5-H*_cis_*), 2.76 (dd, *J* = 14.2, 7.0 Hz, 1H; 4-H_a_ *_trans_*), 2.29 (dd, *J* = 14.4, 1.8 Hz, 1H; 4-H_a_ *_cis_*), 2.19 (dd, *J* = 14.3, 6.6 Hz, 1H; 4-H_b_ *_cis_*), 1.62 (dd, *J* = 14.2, 4.4 Hz, 1H; 4-H_b_ *_trans_*), 1.35 (s, 3H; Me*_trans_*), 1.28 (s, 3H; Me*_cis_*); ^1^H NMR (500 MHz, D_2_O, *cis*/*trans* mixture) δ 5.22 (dd, *J* = 6.9, 4.4 Hz, 1H; 5-H*_trans_*), 5.10 (dt, *J* = 6.3, 1.4 Hz, 1H; 5-H*_cis_*), 2.76 (ddd, *J* = 17.5, 14.2, 7.0 Hz, 1H; 4-H_a trans_), 2.33–2.12 (m, 2H; 4-H *_cis_*), 1.62 (td, *J* = 14.4, 4.4 Hz, 1H; 4-H_b_ *_trans_*), 1.35 (d, *J* = 15.5 Hz, 3H; Me*_trans_*), 1.28 (d, *J* = 15.2 Hz, 3H; Me*_cis_*).; ^13^C NMR (151 MHz, D_2_O) δ 172.7 [*s*, (d, *J* = 2.5 Hz); C-1], 172.3 [*s*, (d, *J* = 3.4 Hz); C-1], 81.1 [*s*, (d, *J* = 1.7 Hz), C-5, *cis*), 80.8 [*d*, (d, *J* = 4.1 Hz); C-5, *trans*], 42.5 [*s*, (d, *J* = 132.4 Hz); C-3], 42.2 [*s*, (d, *J* = 130.2 Hz); C-3], 36.2 [*s*, C-4, *cis*], 35.7 [*s,* C-4, *trans*], 20.3 [*q*, (d, *J* = 2.6 Hz); 3-Me, *trans*], 19.3 [*q*, (d, *J* = 3.3 Hz); 3-Me, *cis*]. ^31^P NMR (202 MHz, D_2_O, *cis*/*trans* mixture) δ 20.8, 19.7. Assignment of *cis*/*trans* configurations was supported by NOESY spectrum of compound **8**.

#### 3.1.3. Chiral Chromatography and Synthesis of Chiral mSF2312

Racemic intermediate **6** was separated by chiral HPLC using a normal phase Lux Cell-1 21.2 × 150 mm (Phenomenex, Torrence, CA, USA) column, with a UV detector set at 254 nm (maximum absorption of the benzyl group). The mobile phase consisted of isocratic 86% Hexane, 9% Ethanol, 4% Isopropanol, 1% acetonitrile, 0.05% TFA; flow rate: 20 mL/min. Analytical runs were performed with ~1 mg of material, while preparatory runs were performed with ~50 mg of material. Chiral purity was verified by re-running on the same column. One volume of deuterated Acetonitrile-d3 was added to provide signal lock for NMR. Each chiral peak yielded a single ^31^P NMR peak, indicating a single *cis*/*trans* diastereomer. This was confirmed by proton NMR. The ^1^H spectrum was obfuscated by mobile phase contaminants, except for the region of around 5 ppm, were benzylic and 5-H protons are visible. Chiral fractions were neutralized with aqueous NaHCO_3_, rotovapped, lyophilized and resuspended in DCM, and the NaHCO3 was filtered out. With 100 mg of starting material intermediate **6**, yield for each peak was ~10 mg. This rather low yield was due to the very conservative collection, to minimize cross-contamination of chiral fractions. Subsequent ethyl and benzyl deprotection were performed in the same way as for racemic MethylSF2312. The identity of MethylSF2312 derived from chirally purified intermediate **6** was verified by NMR and HRMS.

### 3.2. Biology

#### 3.2.1. Enolase Enzymatic Activity

Native lysates of human cell lines were prepared using 20 mM Tris HCl, 1 mM EDTA, and 1 mM β-mercaptoethanol at pH 7.4 as described previously [14]. Enolase activity was measured using two different methods, either by a fluorometric NADH-linked assay or a direct spectrophotometric assay via formation of PEP. In the fluorescent assay, enolase activity was measured via NADH oxidation in a pyruvate kinase–lactate dehydrogenase coupled assay as previously described [4,14]. The assay is conducted in 10 mM KCl, 5 mM MgSO_4_, 100 mM triethanolamine at pH 7.4, with 400 µM NADH and 2 mM ADP. 2-Phosphoglycerate (2-PGA), pyruvate kinase (PK) and lactate dehydrogenase (LDH) are provided in excess, with the conversion of 2-PGA to PEP by enolase being rate limiting. PEP (with ADP) is a substrate of PK; pyruvate formed by this reaction is linked to NADH oxidation by LDH. Enolase activity is determined by measuring oxidation of NADH fluorescently by excitation at 340 nm and emission at 460 nm. The substrate concentration, if not otherwise indicated, was 5 mM 2-PGA. Fluorescence was measured using the Omega Fluorescence Plate Reader (BMG Labtech). Enolase activity was measured in native lysates of *E. coli*. Overnight grown saturated *E. coli* (DH5α) liquid culture was pelleted by centrifugation, washed and resuspended in 50 µL of lysis buffer which contains 150 mM NaCl, 50 mM Tris, 1mM EDTA, 2 mM DTT and 0.025% sodium azide. PMSF (10 mM stock solution) was then added to the lysate to make the final concentration 1 mM with 1% sodium deoxycholate. The suspension was incubated at 4 °C with constant mixing. The extract was centrifuged at 4 °C, 14,000 *g* for 15 min. Enolase activity in *E. coli* native lysates was measured directly by the appearance of PEP from 2-PGA via absorption at 240 nm. The assay medium was the same, except that all the auxiliary reagents (PK/LDH, NADH, ADP) are omitted. Assays were conducted in a 96-well plated format with the direct assay performed in UV-transmissible plates.

#### 3.2.2. Cell Culture

The cell line D423-MG was provided by Dr. Bigner [18]. The 1p36 homozygous deletion in D423-MG spans from the *CAMTA1* to *SLC25A33* genes, including *ENO1*. The generation of isogenic ENO1 and ENO2 ectopically rescued lines was described previously [4,14]. The LN319 cell line was obtained from Cell Bank of The Department of Genomic Medicine at M.D. Anderson. Cells were routinely cultured in high glucose, glutamine, and pyruvate containing Dulbecco’s modified Eagle’s medium (DMEM) with 10% fetal bovine serum (FBS).

#### 3.2.3. Proliferation Assays

Cell proliferation of glioma cell lines was assayed through crystal violet staining. Cells were seeded at 1500 cells/well in 96-well plates, and after 24 h, were treated with dilution series of enolase inhibitors, with each treatment concentration in duplicate wells. At the indicated time, cells were fixed with 10% formalin and stained with crystal violet. Dye extraction was performed using 10% acetic acid solution, and absorbance was read at 595 nm.

#### 3.2.4. Antibiotic Activity Determination using the Disc Diffusion Method

Kirby Bauer disc diffusion antibiotic sensitivity testing was performed on Mueller Hinton II Agar (BD #221275) or BBL™ Columbia Agar with 5% Sheep Blood (BD #221165). An overnight overnight-grown saturated *E. coli* (DH5α) liquid culture was diluted (1:50) in water and streaked on the agar plate to form a bacterial lawn. d-Glucose-6-phosphate (Sigma #G7250) was made in 100 mM stock solution and added to the diluted bacteria to achieve 2 mM final concentration right before streaking the plate. A 7 mm Whatman™ 3MM Chr Chromatography filter paper disc (Whatman # 3030-6189) was imbued with 7 µL of an aqueous solution of inhibitor, placed on the streaked agar plate and incubated overnight at 37 °C.

### 3.3. Structure Determination of ENO2 and Its Inhibitors

Recombinant ENO2 protein was prepared using NiNTA affinity and size exclusion chromatography as described previously [14] Apo crystals of Human Enolase 2 were prepared by streak seeding hanging drop solutions after mixing 0.5 μL 9.1 mg mL^−1^ Enolase 2 with 0.5 μL reservoir solution (200 mM ammonium acetate, 100 mM Bis-Tris and 18–22% (*w*/*v*) PEG 3350). The hanging drops were incubated at room temperature above a 500 μL reservoir. Apo crystals were subsequently soaked overnight in 1 μL drops containing 100 mM Bis-Tis, 200 mM ammonium acetate, 32% (*w*/*v*) PEG 3350 at pH 6.5, supplemented with 2 mM racemate or pure enantiomer of MethylSF2312 compound, prior to flash freezing in liquid nitrogen. X-ray diffraction datasets were collected at 100 K using a wavelength of 1.11587 Å at the Advanced Light Source Beamline 8.3.1, equipped with ADSC Q315r detector. The diffraction images were indexed and integrated using iMOSFLM [19] and scaled using AIMLESS [20]. The X-ray structures were solved by molecular replacement with a Human ENO2 homodimer (PDB code 5EU9) as the search model, using Phenix 1.9-1692 [21]. The monoclinic ENO2:MethylSF2312 crystal structure was iteratively refined using Coot [22] and phenix.refine [23]. The ENO2 structure solved from crystals soaked with the pure enantiomers of MethylSF2312 were both refined using a combination of manual adjustment in Coot and computational refinement with phenix.refine using torsion, libration, screw parameterization [24]. The final structures have good bond lengths are angles with 97.4%, 95.4% and 96.5% of the residues found in the favored regions of the Ramachandran plot for the ENO2 crystals soaked with MethylSF2312 racemate, S-enantiomer form of MethylSF2312 and the R-enantiomer form of MethylSF2312 respectively.

## 4. Conclusions

Our previous work showed that SF2312 is a high potency Enolase inhibitor with potential utility for the treatment of glioblastoma with ENO1 deletions [14]. Structurally, the P-C bond and the phosphonate moiety are generally rare among natural products [25,26]. This makes SF2312 a rather intriguing molecule: in addition to belonging to a rare chemical family, it inhibits glycolysis, a pathway rarely targeted by natural product antibiotics. A question left partially unresolved in our previous manuscript [14] and other publications [4,25] centers on the stereochemistry of the active species. SF2312 has two stereocenters, resulting in four possible enantiomers. The base-labile alpha proton subverted our previous attempts at isolating enantiopure SF2312 [14] (Figure 1).

In this work, we synthesized a C-3 methyl-substituted derivative of SF2312, termed MethylSF2312. The absence of an alpha proton prevents epimerization. As a racemic-diastereomeric mixture, MethylSF2312 exhibited similar and, in some cases, greater potency than SF2312. Successful resolution of the enantiomers of the diethyl (1-(benzyloxy)-5-hydroxy-3-methyl-2-oxopyrrolidin-3-yl)phosphonate precursor allowed us to closely investigate the relationship between stereochemistry and inhibitory activity. Absolute confirmation of each isomer was achieved through a combination of NMR and X-ray analysis. Basic treatment of enantiopure fractions expectedly led to the interconversion of a mixture of the cis and trans anomers. Unlike SF2312, this did not result in racemization due to the absence of an acidic C-3 proton (Supplementary Note 2 in Ref. [14]). (3*S*)-MethylSF2312 showed Enolase inhibitory IC_50_ of ~10 nM while (3*R*)-MethylSF2312 resulted in an IC_50_ ~15 µM. The in vitro Enolase inhibitory activity of each MethylSF2312 enantiomer strongly correlated with the toxicity to Enolase deficient glioma cells and *E. coli*. Finally, a novel X-ray structure of ENO2 co-crystalized with MethylSF2312 unambiguously shows C-3 in the *S*-configuration. The 5′-OH that forms hydrogen bonds with the polar sidechains of Glu167 and His371, strongly suggests a preference for the enantiomer (3*S*,5*S*)-MethylSF2312, which therefore is believed to be the only active species. By analogy, we conclude that (3*S*,5*S*)-SF2312 is the sole active inhibitor of Enolase. Overall, the immense therapeutic potential of the collateral lethality paradigm [4,5] highlights the utility of MethylSF2312 for studying the stereochemical demands of active site Enolase inhibition. Beyond cancer, the significance of exploiting glycolytic vulnerabilities across various infectious diseases [4,7,8,9] is supported by the initial finding on the antimicrobial effects of SF2312 under anaerobic conditions [25]: that structural differences between the Trypanosoma and human Enolase exist and may present an opportunity for selective inhibition of the former over the latter. This is further substantiated by the uptake of phosphonate compounds through the G6P shuttle present in certain microorganisms (Figure 3; note the profound effect by MethylSF2312 sensitivity with the addition of G6P, which induces expression of the G6P transporter to increase permeation of MethylSF2312). Together, these data support the significance in defining the 3*S* geometric requirements for antimicrobial and anticancer activity, through analysis of the Enolase inhibition effects by MethylSF2312.

## Figures and Tables

**Table 1:** X-ray diffraction data collection and refinement statistics. Values in parentheses are for the highest resolution shell.

	Enolase 2: MethylSF2312 (PDB 5EU9)	Enolase 2:*S*-MethylSF2312 (PDB 5TIJ)	Enolase 2: *R*-MethylSF2312 (PDB 5TD9)
Data Collection			
Wavelength (Å)	1.116	1.116	1.116
Space group	P1 2_1_ 1	P2_1_ 2_1_ 2_1_	P2 2_1_ 2_1_
Cell dimensions			
a, b, c (Å)	119.3, 110.3, 136.9	68.25, 108.38, 117.48	68.32, 112.87, 119.92
α, β, γ (°)	90, 90, 90	90, 90, 90	90, 90, 90
N°. of unique reflections	217,213	26,354	40,898
Resolution (Å)	89.93–2.05 (2.08–2.05)	59.01–2.63 (2.76–2.63)	50.00–2.31 (2.35–2.31)
Rmerge (all I^+^ and I^−^)	0.200 (0.994)	0.110 (0.290)	0.110 (0.391)
I/σI	6.4 (2.0)	11.7 (5.2)	32.2 (5.3)
Completeness (%)	97.6 (91.7)	99.6 (98.2)	99.8 (96.4)
Redundancy	6.7 (6.4)	6.5 (6.1)	8.0 (7.3)
Refinement			
Resolution (Å)	89.93–2.05	59.01–2.63	43.51–2.32
σF	1.34	1.38	1.34
N°. of reflections	217,011	26,306	40,812
R_work_/R_free_	0.154/0.192	0.206/0.267	0.200/0.245
Wilson B	21.4	30.0	33.4
N°. of atoms			
Protein	26,693	6627	6644
Ligands	164	26	-
Ions	16	2	4
Water	2238	110	86
Average B-factors (Å^2^)			
Protein	20.8	37.6	54.4
Ligands	23.9	33.6	-
Ions	14.7	34.2	68.0
Water	28.7	28.2	43.1
r.m.s.d.			
Bond lengths (Å)	0.012	0.005	0.006
Bond angles (°)	1.039	0.451	0.636
